# Investigating Linkages Between Spatiotemporal Patterns of the COVID-19 Delta Variant and Public Health Interventions in Southeast Asia: Prospective Space-Time Scan Statistical Analysis Method

**DOI:** 10.2196/35840

**Published:** 2022-08-09

**Authors:** Wei Luo, Zhaoyin Liu, Yuxuan Zhou, Yumin Zhao, Yunyue Elita Li, Arif Masrur, Manzhu Yu

**Affiliations:** 1 Department of Geography National University of Singapore Singapore Singapore; 2 Department of Civil and Environmental Engineering National University of Singapore Singapore Singapore; 3 Department of Earth, Atmospheric, and Planetary Sciences Purdue University West Lafayette, IN United States; 4 Department of Geography Pennsylvania State University State College, PA United States

**Keywords:** COVID-19, Delta variant, space-time scan, intervention, Southeast Asia

## Abstract

**Background:**

The COVID-19 Delta variant has presented an unprecedented challenge to countries in Southeast Asia (SEA). Its transmission has shown spatial heterogeneity in SEA after countries have adopted different public health interventions during the process. Hence, it is crucial for public health authorities to discover potential linkages between epidemic progression and corresponding interventions such that collective and coordinated control measurements can be designed to increase their effectiveness at reducing transmission in SEA.

**Objective:**

The purpose of this study is to explore potential linkages between the spatiotemporal progression of the COVID-19 Delta variant and nonpharmaceutical intervention (NPI) measures in SEA. We detected the space-time clusters of outbreaks of COVID-19 and analyzed how the NPI measures relate to the propagation of COVID-19.

**Methods:**

We collected district-level daily new cases of COVID-19 from June 1 to October 31, 2021, and district-level population data in SEA. We adopted prospective space-time scan statistics to identify the space-time clusters. Using cumulative prospective space-time scan statistics, we further identified variations of relative risk (RR) across each district at a half-month interval and their potential public health intervention linkages.

**Results:**

We found 7 high-risk clusters (clusters 1-7) of COVID-19 transmission in Malaysia, the Philippines, Thailand, Vietnam, and Indonesia between June and August, 2021, with an RR of 5.45 (*P*<.001), 3.50 (*P*<.001), 2.30 (*P*<.001), 1.36 (*P*<.001), 5.62 (*P*<.001), 2.38 (*P*<.001), 3.45 (*P*<.001), respectively. There were 34 provinces in Indonesia that have successfully mitigated the risk of COVID-19, with a decreasing range between –0.05 and –1.46 due to the assistance of continuous restrictions. However, 58.6% of districts in Malaysia, Singapore, Thailand, and the Philippines saw an increase in the infection risk, which is aligned with their loosened restrictions. Continuous strict interventions were effective in mitigating COVID-19, while relaxing restrictions may exacerbate the propagation risk of this epidemic.

**Conclusions:**

The analyses of space-time clusters and RRs of districts benefit public health authorities with continuous surveillance of COVID-19 dynamics using real-time data. International coordination with more synchronized interventions amidst all SEA countries may play a key role in mitigating the progression of COVID-19.

## Introduction

COVID-19 is a global epidemic caused by SARS-CoV-2. SARS-CoV-2 is highly contagious as it easily spreads across humans, animals, and the environment via contact, droplets, air, fomites, and other transmission modes [1]. In December 2019, COVID-19 was first identified in Wuhan, a transportation and communication hub located in central China, and rapidly spread to surrounding regions in China. Although governments took a certain level of precautions and control regulation, international cases managed to emerge along with significant outbreaks worldwide. As of December 12, 2021, the disease had infected over 200 million people worldwide [2].

Countries in Southeast Asia (SEA) saw unprecedented challenges in health and social care systems, tourism, trade, and the service industry with the outbreak of COVID-19 [3]. Many countries in SEA made efforts to recover the economy during late 2020 and early 2021 by progressively easing lockdowns and strengthening export orders, but they were hammered by the wave of the new COVID-19 Delta variant [4]. The Delta variant is estimated to be 2 or even 4 times more transmissible than the original virus, with a reproductive number (R0) over 5, which means that more than 5 people can be further infected by each infected individual [5]. Since April 2021, SEA observed an exponential increase in new cases due to the Delta variant and has become an emerging hotspot of COVID-19 [6]. This new outbreak of the Delta variant heavily burdened national and international business in SEA. Interruption of the supply chain of many products (eg, garments, automobile parts, semiconductors) had a substantial impact on the manufacturing industries in SEA, especially for countries relying on them (ie, Vietnam, Malaysia, Thailand, Indonesia, and the Philippines) [4]. Due to these economic pressures, a growing number of countries in SEA planned to live with the virus and adjusted their public health intervention policies. [7]. Under this circumstance, monitoring outbreaks and identifying the space-time clusters of infection have become significant for a coordinated response to the epidemic in SEA.

Spatiotemporal analysis has been widely used in research of COVID-19 propagation to illustrate the characteristics and mechanism of COVID-19 spatial propagation. It can provide public health authorities with important information about the pandemic to enable better management under the situation [8-10]. Among diverse spatiotemporal methods, space-time scanning is one of the most popular methods adopted by many studies to explore spatiotemporal clusters in different regions worldwide, such as Mainland China [11], the United States [12], Mexico [13], Spain [14], Malaysia [15], Bangladesh [16], Brazil [17], and South Korea [18]. In SEA, previous studies have applied this analysis to investigate the first wave of COVID-19 cases [19,20]. However, these studies have mainly focused on each individual country without exploring propagation patterns and progression characteristics with collective public health interventions at the regional scale. Previous research has shown that regional coordination could interrupt the transmission of COVID-19 in an effective way [10,17,21-23]. To contain the emerging epidemic of COVID-19 and minimize its risk, countries in SEA have deployed various preventive and containment measures, such as lockdowns, social distancing restrictions, and mandatory tracking and trace methods [24,25].

Delta variant transmission has shown significant spatial heterogeneity in SEA because different countries adopted different interventions as the virus spread. Hence, this paper aims to identify the space-time clusters of outbreaks of COVID-19 caused by the SARS-CoV-2 Delta variant in SEA. We utilized district-scale daily confirmed cases of 7 SEA countries from June to October 2021 to identify the active and emerging clusters of the disease and summarized relative policies to investigate the potential linkage between government interventions and pandemic progression. Our work will contribute to regional surveillance of COVID-19 progression in SEA and provides essential information about COVID-19 propagation to public health authorities, which is beneficial for timely policymaking according to the dynamic COVID-19 situation.

## Methods

### Study Areas and Relevant Interventions

Our study focused on the COVID-19 Delta variant in SEA. Due to the constraint of relevant data availability, we were only able to include 7 countries, namely (1) Indonesia, (2) Malaysia, (3) the Philippines, (4) Singapore, (5) Thailand, (6) Vietnam, and (7) Brunei, as they disclosed data of daily confirmed cases at a district level. During the second COVID-19 outbreak caused by the Delta variant, the dynamic of interventions implemented by different countries along the way may have caused a fluctuation in transmission. For example, Thailand, Singapore, Malaysia, the Philippines, and Vietnam began to relax their restrictions around August 2021, which may have caused significant changes in the pandemic patterns (Table 1). The diverse policies will help explain the progression and transmission of the Delta variant of COVID-19 in the following analysis. The interventions were aggregated by the Center for Strategic & International Studies (CSIS) [26].

**Table 1:** Major public health interventions in SEA^a,b^.

Duration		Interventions
**Indonesia**		
	June 1-14, 2021	Micro community activity restrictions (*Pemberlakuan Pembatasan Kegiatan Masyarakat* [PPKM] in Indonesian) implemented, which include guidance on travel, work-from-home policies, online teaching, the restaurant industry, and gatherings
	June 14-28, 2021	Community activity restrictions (ie, the PPKM) extended
	July 2-20, 2021	Emergency public activity restrictions implemented across Java and Bali
	July 7-September 20, 2021	The PPKM extended covering the entire country
	August 31-September 6, 2021	COVID-19 restrictions relaxed
	September 7-October 31, 2021	COVID-19 restrictions eased for tourists across most of Java
	October 5-18, 2021	Community restrictions extended in Java and Bali
	October 19-31, 2021	PPKM restrictions eased to level 2 in Jakarta and Tangerang
**Thailand**		
	July 17-September 30, 2021	Nationwide emergency imposed
	August 1-31, 2021	Tighter restrictions imposed, including travel curbs, curfews, and travel from other regions
	August 16-30, 2021	Lockdown measures extended for 2 weeks
	August 23-October 31, 2021	The country’s strategy to shifted “learning to live with COVID-19” by relaxing some restrictions and reopening its borders to vaccinated visitors gradually
	September 1-October 31, 2021	Domestic flights from and to Bangkok and other high-risk areas allowed to resume
	October 1-31, 2021	Restrictions in dark-red provinces (highest-risk regions) eased
	October 16-31, 2021	Curfew shortened
**Singapore**		
	June 21-July 22, 2021	Indoor dining resumed
	July 22-August 8, 2021	Returned to phase 2 (heightened alert) status, putting in place enhanced restrictions, including limiting social gatherings to 2 people and banning indoor and outdoor dining
	August 8-September 27, 2021	Restrictions relaxed for fully vaccinated people
	August 19-September 27, 2021	Workforce allowed to return to their offices
	August 20-October 31, 2021	Border restrictions eased
	September 14-October 31, 2021	Nationwide booster shot campaign further strengthened
	September 27-October 31, 2021	In-person gathering limited from 5 to 2 people but border restrictions further eased for fully vaccinated people
**Malaysia**		
	June 1-28, 2021	Nationwide lockdown implemented
	July 3-September 14, 2021	Lockdowns in Kelantan, Pahang, Perak, Perlis, and Terengganu relaxed
	August 1-October 31, 2021	Extending the country’s state of emergency ended
	August 2-September 14, 2021	Restrictions further loosened in Perlis, Sarawak, and Labuan
	August 8-September 14, 2021	Some restrictions relaxed for fully vaccinated people in 8 states
	August 21-September 14, 2021	Social distancing measures loosened for outdoor sports and in-person dining for fully vaccinated people
	September 10-14, 2021	Travel, dining, and tourism restrictions relaxed in Kuala Lumpur, Selangor, and Putrajaya
	September 9-October 31, 2021	Creative industry reopened
	September 14-October 1, 2021	COVID-19 lockdown restrictions further eased
	October 1-31, 2021	Movement restrictions relaxed
**The Philippines**		
	June 1-30, 2021	Travel restrictions extended on inbound travelers coming from India and 6 other countries
	June 29-July 15, 2021	Movement restrictions in the capital and surrounding provinces extended
	July 25-31, 2021	Travel from Malaysia and Thailand suspended, restrictions in the Manila area tightened
	August 13-31, 2021	Ban on travelers from India, Pakistan, Bangladesh, Sri Lanka, Nepal, the UAE, Oman, Thailand, Malaysia, and Indonesia extended
	August 6-20, 2021	Reverted to the strictest level of lockdown in Metro Manila
	August 21-31, 2021	COVID-19 restrictions eased in the Manila capital region
	September 7-15, 2021	Movement restrictions extended in Manila
	September 16-October 1, 2021	Wide-scale restrictions eased in Manila despite direct warnings from the World Health Organization (WHO) against reopening certain businesses
	October 1-31, 2021	Movement restrictions eased in the Manila capital region
	October 13-31, 2021	Curfew hours shortened in Metro Manila
	October 16-31, 2021	Alert level lowered in the National Capital Region from level 4 to 3
**Vietnam**		
	June 14-30, 2021	Social distance measures extended in Ho Chi Minh City
	July 7-21, 2021	2-week lockdown implemented in Ho Chi Minh City
	July 18-August 1, 2021	2-week lockdown imposed in 16 southern provinces
	August 15-September 15, 2021	Social distancing requirements extended in Ho Chi Minh City
	September 16-30, 2021	COVID-19 restriction extended in Ho Chi Minh City
	September 23-October 31, 2021	Lockdown restrictions eased in several provinces
	October 1-31, 2021	Select economic activities resumed in Ho Chi Minh City
	October 13-31, 2021	Coach buses to resume operations allowed in Ho Chi Minh City between the city and nearby provinces
	October 15-31, 2021	Risk level reduced in Ho Chi Minh City
**Brunei**		
	August 8-October 3, 2021	COVID-19 restrictions implemented
	September 1-15, 2021	Travel restrictions to and from India, Nepal, Sri Lanka, Pakistan, and Bangladesh extended
	October 4-17, 2021	Movement restrictions tightened
	October 13-31, 2021	Nightly curfew extended

^a^SEA: Southeast Asia.

^b^Note that up to October 31, 2021, some of the interventions were still continuously effective. We therefore defined the ending date of the duration of such interventions as the last day of our study period.

### COVID-19 Daily Cases and Populations

**Figure 1:**
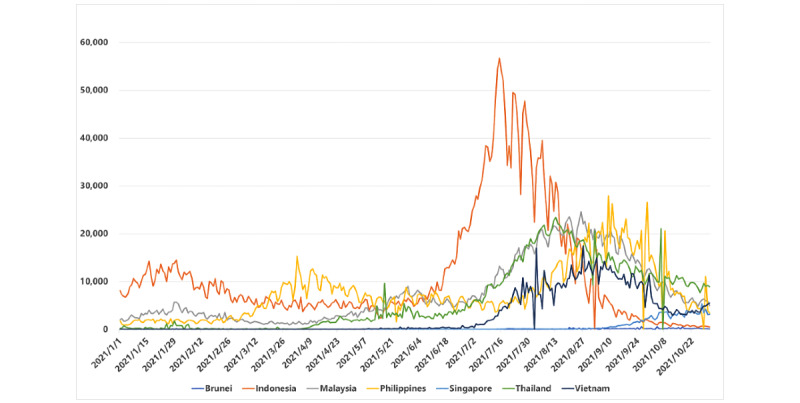
Daily confirmed new COVID-19 cases in Southeast Asia (SEA) from January 1 to October 31, 2021.

We obtained or extracted data of COVID-19–confirmed cases from the official websites of public health authorities in the 7 countries and Johns Hopkins University's Center for Systems Science and Engineering GIS dashboard (Table 2). From March to May 2021, the 7 countries in SEA successively identified the Delta variant, which dominated mass infections in the next few months (Table 3) [27-33]. Figure 1 shows the substantial growth of confirmed cases of COVID-19 in the 7 countries from June 2021, and most of the countries experienced apparent fluctuations of daily confirmed cases from June to October 2021. Therefore, we adopted data from June 1 to October 31, 2021, which is the approximate date of the second COVID-19 outbreak in these 7 countries in SEA. We aggregated the data at the first administrative level, except for those in Singapore and Brunei, which were aggregated at the country level, considering the similar magnitude of area and population in each analytic unit. We obtained or extracted population data from statistical reports and yearbooks from those countries (Table 2).

**Table 2:** Collected data and their sources.

Country	Case source	Population source
Indonesia	KAWALCOVID19 and the National Board of Confirmed Case Development [34]	Statistics Indonesia [35]
Malaysia	Official data on the COVID-19 epidemic in Malaysia [36]	Department of Statistics Malaysia [37]
The Philippines	Department of Health, the Philippines [38]	Philippine Statistics Authority [39]
Singapore	Ministry of Health, Singapore [40]	Department of Statistics, Singapore [41]
Thailand	Ministry of Public Health, Department of Disease Control Situational Reports [42]	National Statistical Office of Thailand [43]
Brunei	Johns Hopkins University’s Center for Systems Science and Engineering COVID-19 data [44]	Department of Economic Planning and Statistics [45]
Vietnam	Ministry of Health, Vietnam [46]	General Statistics Office of Vietnam [47]

**Table 3:** Month of identification of the Delta variant in 7 countries.

Country	Month of first confirmed case of Delta variant
Indonesia	March 2021
Thailand	May 2021
Singapore	April 2021
Malaysia	May 2021
The Philippines	May 2021
Vietnam	April 2021
Brunei	August 2021

### Space-Time Scan Statistical Analysis

To explore emerging and active space-time clusters of COVID-19 cases in SEA, we conducted prospective space-time scan statistical analysis using SaTScan version 9.6 [48], which is often used to detect spatial clusters of infectious diseases [49,50]. Using space-time scan statistics, we identified and mapped significant clusters of the Delta variant in SEA, considering the uneven distribution of population size. The space-time scan statistics adopted a cylinder to detect potential space-time clusters in SEA, which can cover each possible location, size, and period [51]. For each cylinder, the base represented space, the height represented time, and the center represented the centroids of study units throughout SEA. The size of the cylindrical window was expanded until reaching specific maximum spatial and temporal upper bounds, which were set to 10% of the population risk and 50% of the study period, respectively, in this study. We set the minimum duration of each cluster to 2 days for surveillance of the continuously existing clusters. The minimum number of cases in each cluster was set to 3 in order to ensure that there must be at least 3 cases in each cluster.

We assumed that the COVID-19 cases follow a Poisson distribution according to the population of study units in SEA. The null hypothesis (*H_0_*) indicates that the model reflects infection of COVID-19 having a constant intensity *μ* within or outside the cylinder, which is proportional to the at-risk population. The alternative hypothesis (*H_A_*) indicates that the observed cases are more than expected cases, which reflects an increased risk within a cylinder. Expected cases were calculated by Equation (1) [12]:



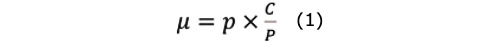



where *p* represents the population within a study unit, *C* represents the total COVID-19 cases in our study area (ie, 7 countries in SEA), and *P* represents the total estimated population within our study area.

A maximum likelihood ratio test was performed to evaluate the null and alternative hypotheses. It identified scanning windows with an elevated risk for COVID-19, which was defined by Equation (2) [17,52]:



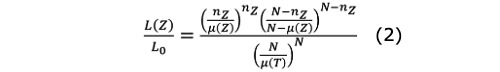



where *L(Z)* represents the likelihood function for cylinder *Z*, *L_0_* represents the likelihood function for *H_0_*, *n_Z_* represents the number of COVID-19 cases in a cylinder, *μ(Z)* represents the number of expected cases in cylinder *Z*, and N represents the total number of observed cases for the 7 countries in SEA across all periods. When the likelihood ratio is greater than 1, there is an elevated risk in the cylinder, and the cylinder with the maximum likelihood ratio should be the most likely cluster.

The relative risk (RR) of COVID-19 was assumed homogeneous throughout different districts within the same cluster. To make the results more reasonable, we calculated the RR for each study unit within a cluster to explore the spatial heterogeneity of the RR of COVID-19, as given by Equation (3) [53]:



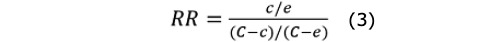



where *c* represents the total number of COVID-19 in a study unit, *e* represents the total number of expected cases in a study unit, and *C* represents the total number of observed cases in the 7 countries of SEA. The formula indicates that the RR represents the estimated risk in a study unit, divided by the risk outside that unit. Specifically, if a location (cluster or study unit) has an RR of 3, the population within the location is three times more likely to be exposed to COVID-19 infection than its outside. The high-risk clusters are characterized by higher observed than expected COVID-19 cases (RR>1), while the low-risk clusters are characterized by higher expected than observed COVID-19 cases (RR<1).

The following sections reveal significant emerging clusters of COVID-19 cases in 7 countries of SEA from June 1 to October 31, 2021. Considering that some SEA countries (eg, Malaysia, the Philippines, Singapore, Thailand) started loosening their restrictions from August 2021, along with strengthening their vaccination plan, we divided the timeline into 2 parts (ie, June 1-August 31, 2021, and June 1-October 31, 2021) in order to identify the dynamics of the clusters. Additionally, we explored the variation in the RR across each district in each half-month, which is approximately equal to the incubation period of an infection [54], using a cumulative half-month prospective scanning approach. Next, we compared the interventions with the discovered space-time characteristics to identify the potential linkage between political intervention and the progression of the Delta variant of COVID-19.

## Results

### Dynamics of District-Level Merging Clusters in SEA

#### Results From June 1 to August 31, 2021

**Figure 2:**
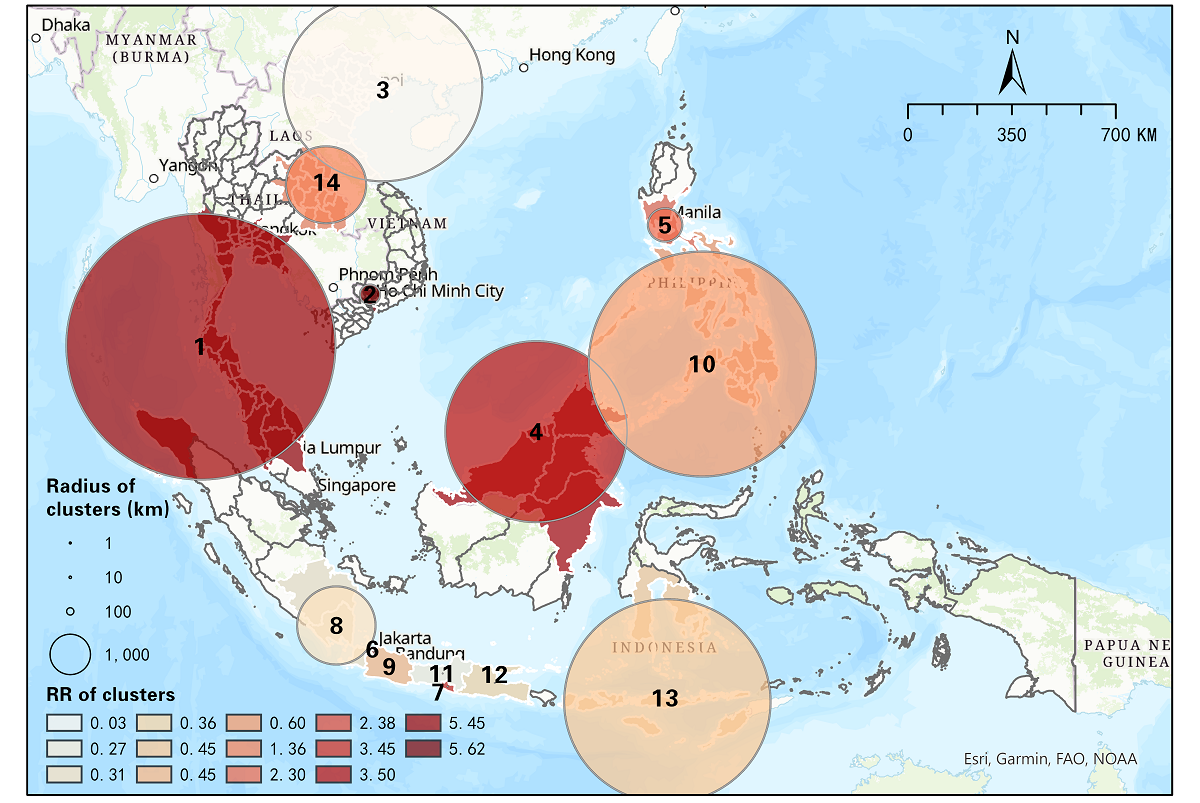
Spatial distribution of space-time clusters of COVID-19 from June 1 to August 31, 2021. RR: relative risk.

As shown in Table 4, 14 significant space-time clusters were identified from June 1 to August 31, 2021, in SEA, including 7 (50%) high-risk clusters (RR>1) and 7 (50%) low-risk clusters (RR<1). Most of the high-risk clusters emerged between mid-July and late August 2021, which means the situation of COVID-19 in SEA became severe during this period. Specifically, cluster 1 was the most likely, and a transnational cluster, containing 39 (83%) high-risk districts (RR>1) out of 47 districts of Malaysia and Thailand. This cluster had the highest RR of 5.45, which means people in this cluster were 5.45 times more likely to be exposed to COVID-19 than in other regions. Similarly, cluster 2 had an RR of 5.62 and contained 2 districts of Vietnam, namely Binh Duong and Ho Chi Minh City. Another transnational cluster was cluster 4, with an RR of 3.50, containing 6 districts of Malaysia, Indonesia, and Brunei. Additionally, north Thailand and the north Philippines also emerged as high-risk clusters from July 22 to August 31, 2021, and from August 11-31, 2021, respectively. There were also 2 clusters emerging in Indonesia, and they contained only 1 district, which was Jakarta, with an RR of 2.38, and Daerah Istimewa Yogyakarta (DIY), with an RR of 3.88. In addition, 3 high-risk clusters in Indonesia revealed that the population within a number of regions in Indonesia was more likely to be exposed to COVID-19 compared to other regions in SEA during this period. Additionally, there were 7 low-risk clusters distributed across other regions of SEA (eg, north of Vietnam, south of the Philippines, and some other districts of Indonesia), which means the population within these clusters was less likely to be exposed to COVID-19. Note that cluster 10, with an RR of 0.60, contained 2 high-risk districts of the Philippines (ie, region VII, with an RR of 1.07, and region X, with an RR of 1.08). Figure 2 shows the distribution of each cluster. There were a number of small-scale clusters in south Indonesia, while the largest-scale cluster appeared across southern Thailand and north Malaysia. From the results, the Delta variant of COVID-19 had a wider influence in Malaysia and Indonesia in the early phase, while some regions in Vietnam and the Philippines had relatively high risk as well.

**Table 4:** Space-time clusters of COVID-19 from June 1 to August 31, 2021.

Cluster	Duration (days)	Total districts, N	*P* value	Observed	Expected	RR^a^	Districts (RR>1), n (%)
1	July 17-August 31	47	<.001	1,246,176	278,375.06	5.45^b^	39 (83)
2	July 18-August 31	2	<.001	300,418	55,874.66	5.62^b^	2 (100)
3	July 17-August 31	30	<.001	6335	223,562.03	0.03	0
4	July 22-August 31	6	<.001	184,097	53,834.73	3.50^b^	4 (67)
5	August 11-31	3	<.001	196,693	87,355.91	2.30^b^	3 (100)
6	July 17-August 31	1 (Jakarta)	<.001	123,567	52,481.33	2.38^b^	1 (100)
7	July 17-August 31	1 (DIY^c^)	<.001	62,476	18,229.28	3.45^b^	1 (100)
8	August 7-31	3	<.001	24,861	79,338.99	0.31	0
9	August 7-31	1 (Jawa Barat)	<.001	59,194	130,362.28	0.45	0
10	July 17-August 31	11	<.001	158,665	259,719.07	0.60	2 (18)
11	August 18-31	1 (Jawa Tengah)	<.001	15,132	55,221.58	0.27	0
12	August 15-31	1 (Jawa Timur)	<.001	26,939	74,674.85	0.36	0
13	August 15-31	4	<.001	18,415	41,030.60	0.45	0
14	July 22-August 31	14	<.001	82,443	60,927.09	1.36^b^	11 (79)

^a^RR: relative risk.

^b^High-risk clusters.

^c^DIY: Daerah Istimewa Yogyakarta.

#### Results From June 1-October 31, 2021

**Figure 3:**
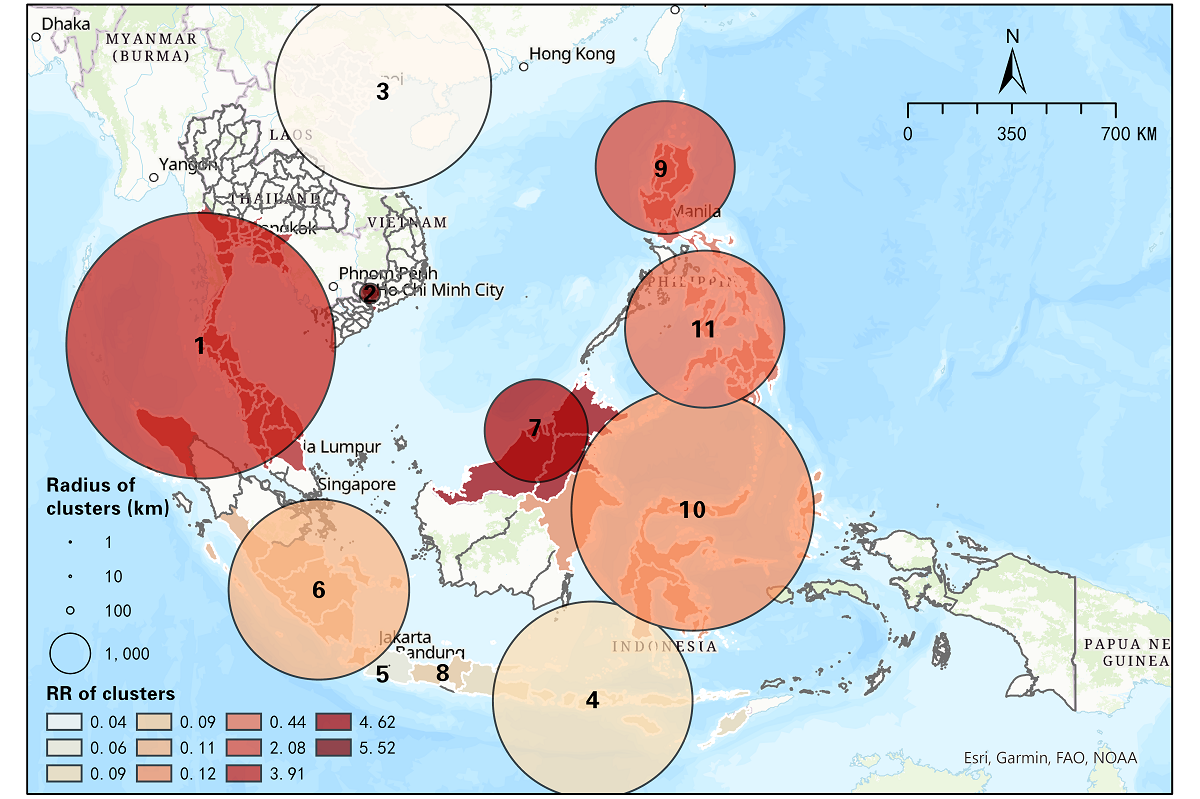
Spatial distribution of space-time clusters of COVID-19 from June 1 to October 31, 2021. RR: relative risk.

In total, 11 significant clusters were detected from June 1 to October 31, 2021, which was 3 less than the early phase. Among the 11 clusters, there were only 4 (36%) high-risk clusters (RR>1), decreasing from 7 in the previous period (Table 5). The most likely cluster was the same as in the previous period, which covered partial districts of Thailand and Malaysia. The RR of cluster 1, however, decreased from 5.45 to 3.91 in this period, while the number of high-risk districts increased from 39 to 45, which implies that more districts were affected by COVID-19. Cluster 2 was also the same as in the previous period, containing Binh Duong and Ho Chi Minh City. In addition, cluster 7 evolved from cluster 4 in the previous period, with the exclusion of Kalimantan Timur. The RR of this cluster increased from 3.50 to 4.62, indicating that people in this cluster were more likely to be infected in this period. Similarly, the Cordillera Administrative Region, region II, and region I merged with 1 high-risk cluster in the north Philippines and formed a larger cluster. The rest of the clusters were low-risk clusters with RR<1. Figure 3 visualizes the distribution of clusters in this period, which shows directly that some of the clusters remained between 2 continuous periods, while a number of clusters in the previous period disappeared and some new clusters appeared in this period. Especially in Indonesia, high-risk clusters in Jakarta and DIY disappeared, which emerged as low-risk clusters with other districts. Overall, the space-time scan statistic results show the transmission and dispersal of the Delta variant of COVID-19 in SEA from 2 different periods.

**Table 5:** Space-time clusters of COVID-19 from June 1 to October 31, 2021.

Cluster	Duration (days)	Total districts, N	*P* value	Observed	Expected	RR^a^	Districts (RR>1), n (%)
1	August 17-October 31	47	<.001	1,441,175	421,090.26	3.91^b^	45 (96)
2	August 17-October 31	2	<.001	456,029	86,398.28	5.52^b^	2 (100)
3	August 17-October 31	33	<.001	13,724	355,228.49	0.04	0
4	August 28-October 31	4	<.001	34,492	357,601.25	0.09	4 (100)
5	August 27-October 31	1 (Jawa Barat)	<.001	19,419	315,097.51	0.06	0
6	August 27-October 31	8	<.001	37,326	342,621.73	0.11	0
7	August 17-October 31	5	<.001	283,690	63,058.92	4.62^b^	4 (80)
8	August 21-October 31	1 (Jawa Tengah)	<.001	24,989	260,017.41	0.09	0
9	August 17-October 31	6	<.001	728,003	366,113.53	2.08^b^	6 (100)
10	September 3-October 31	8	<.001	17,783	145,558.63	0.12	0
11	October 4-31	10	<.001	60,614	136,535.58	0.44	0

^a^RR: relative risk.

^b^High-risk clusters.

### Temporal Progression of the RR of COVID-19 in SEA

**Figure 4:**
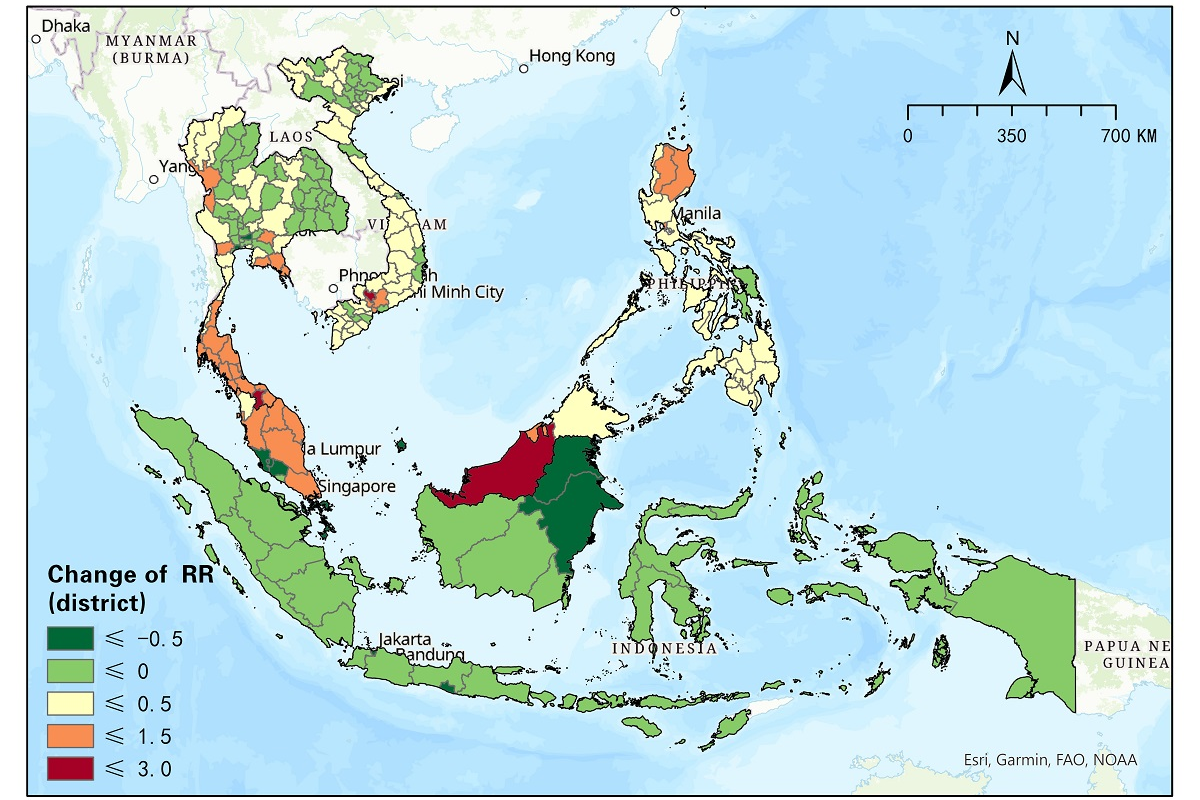
Changes in the relative risk (RR) of COVID-19 (district level) between two periods (June 1-August 31 and June 1-October 31, 2021).

Figure 4 presents changes in the district RR of COVID-19 in SEA between 2 outbreak periods, June 1-August 31 and June 1-October 31, 2021. Overall, this temporal change in the RR manifests different space-time characteristics in terms of COVID-19 progression before and after the countries changed their intervention strategies in handling COVID-19 in July and August 2021. Among the 7 countries in SEA, Indonesia was the only one that showed an overall positive trend of a decreasing RR in every district, while alarming changes of an increasing RR were frequently seen in the other 6 countries, especially in Singapore, the Philippines, Malaysia, and Vietnam.

Specifically, all districts of Indonesia observed a decrease in the RR to different extents. Although the RR in most districts of Indonesia slightly decreased by 0.05-0.5, the other 5 districts (ie, Jakarta, DIY, Kalimantan Utara, Kalimantan Timur, and Kepulauan Riau) manifested a rather significant decrease (≤–0.5), in which the highest difference (–1.4) between the 2 periods was seen in Jakarta, the capital of Indonesia. Note that Jakarta was one of the major emerging risk districts early in the second outbreak and still faced a relatively high RR (2.79) until the end of our study period. On the contrary, the RR of all districts in the Philippines increased between the 2 outbreak periods, indicating an overall deterioration in the risk impact of COVID-19. Fortunately, among a total of 17 districts in the Philippines, 14 (82%) showed minor increases (≤0.5). The other 3 districts, namely the National Capital Region, region II, and the Cordillera Administrative Region, manifested an increase from 0.59 to 1.23. Meanwhile, no significant increase (>1.5) was observed, indicating that the most severe variation in the RR did not occur in the Philippines.

Other countries including Singapore, Malaysia, Brunei, Thailand, Vietnam, in contrast, showed a variety of RR changes in different districts, approximately half of which (n=39, 51%) showed increases in the RR, and half decreased among the 77 districts in Thailand. It is obvious that coastal areas in the south of the country faced a more elevated RR than inland areas in the central, eastern, and northern districts. The RR in almost all districts in Vietnam slightly changed, ranging from –0.08 to 0.28, except for 3 connected cities (ie, Dong Nai: 0.63, Ho Chi Minh City: 0.72, and Binh Duong: 1.73). As for Malaysia, 10 (63%) of 16 districts showed an increase in the RR and took up a major proportion of the country. Labuan and Kuala Lumpur observed the most obvious decrease among SEA districts. It should be noted that they were also the districts most severely threatened by COVID-19 risks early in the study period (RR>10) and had the relatively highest RR in SEA until the end of the study. Additionally, Singapore saw an increased RR of 1.43. The RR in Brunei also increased but still remained less than 1.

**Figure 5:**
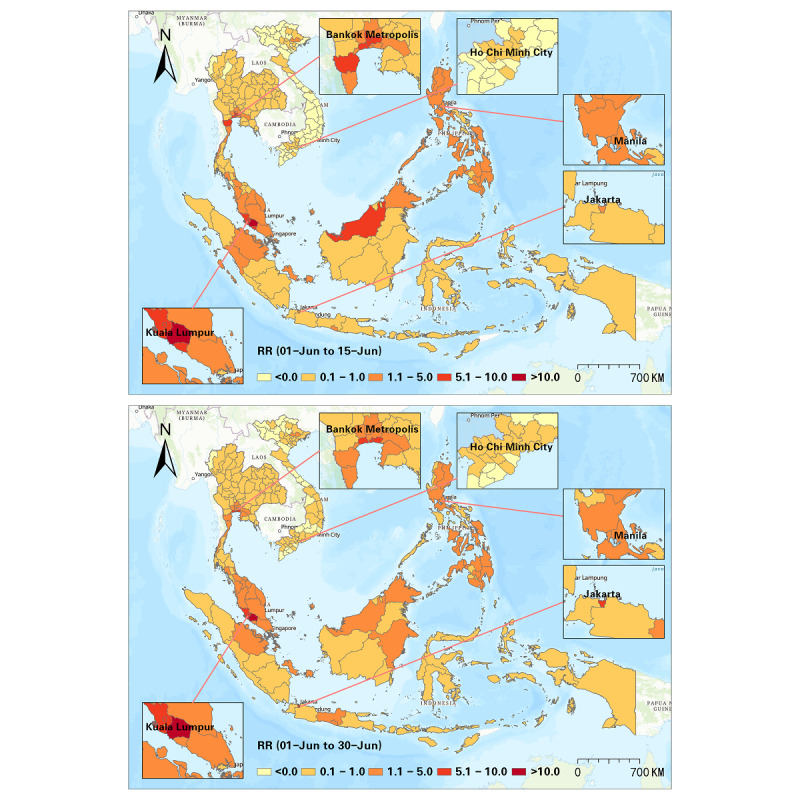
Spatial patterns of progression of the COVID-19 RR in SEA (June 1-15 and June 1-30, 2021). RR: relative risk; SEA: Southeast Asia.

**Figure 6:**
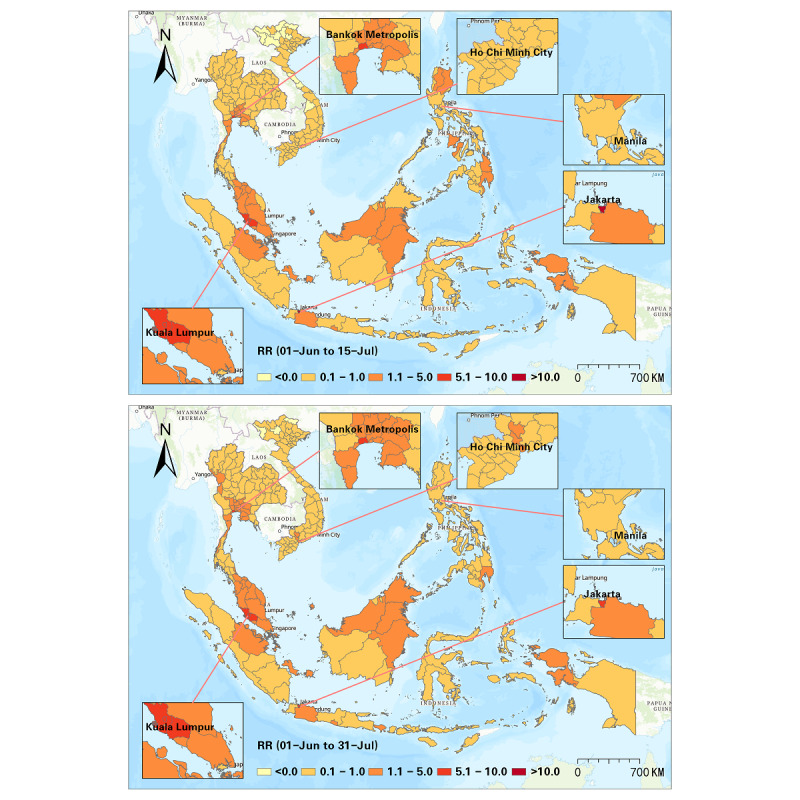
Spatial patterns of progression of the COVID-19 RR in SEA (June 1-July 15 and June 1-July 31, 2021). RR: relative risk; SEA: Southeast Asia.

**Figure 7:**
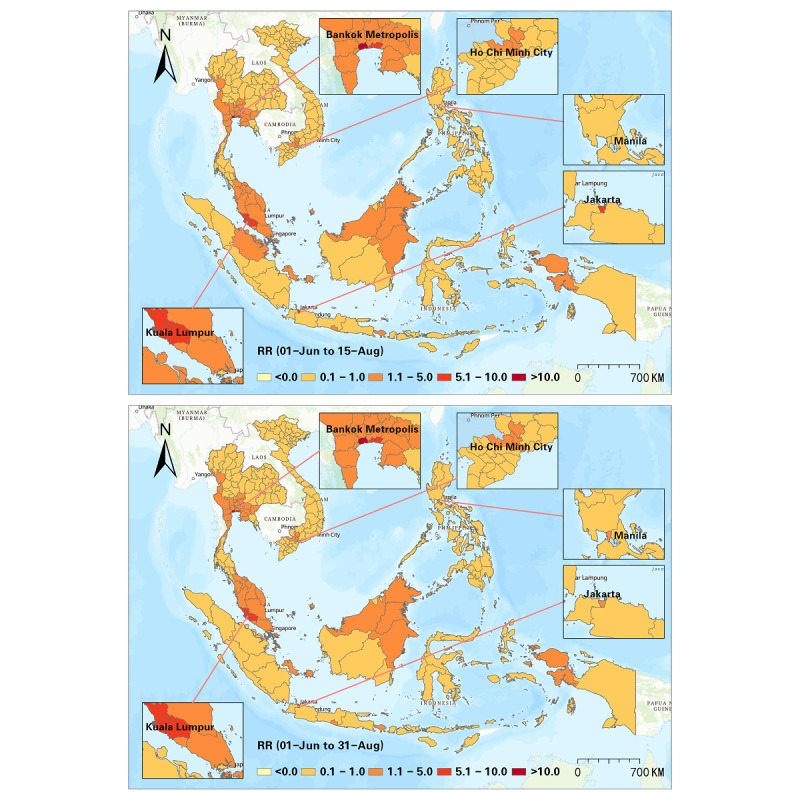
Spatial patterns of progression of the COVID-19 RR in SEA (June 1-August 15 and June 1-August 31, 2021). RR: relative risk; SEA: Southeast Asia.

**Figure 8:**
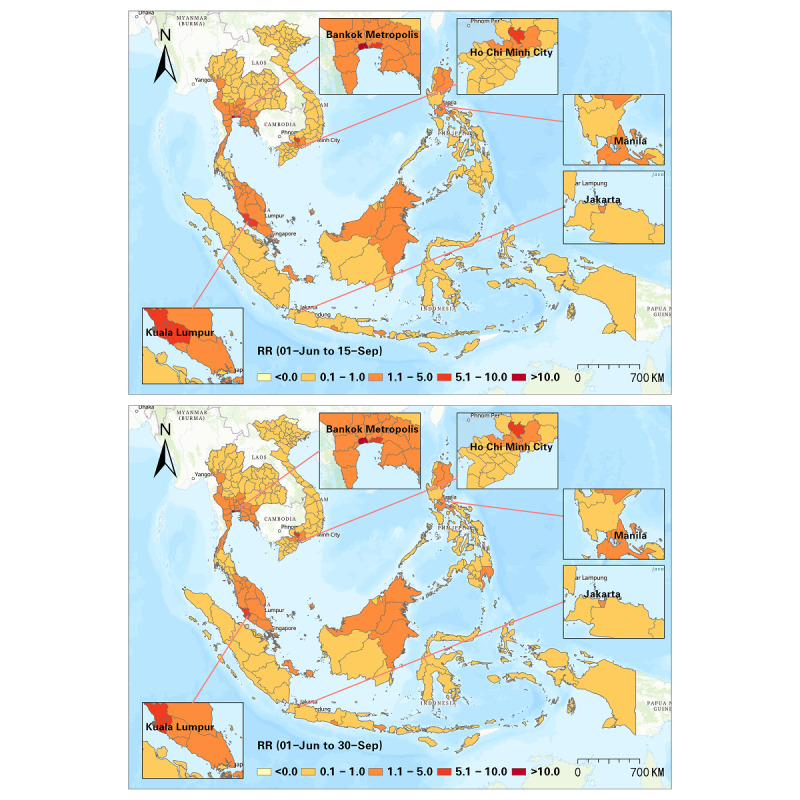
Spatial patterns of progression of the COVID-19 RR in SEA (June 1-September 15 and June 1-September 30, 2021). RR: relative risk; SEA: Southeast Asia.

**Figure 9:**
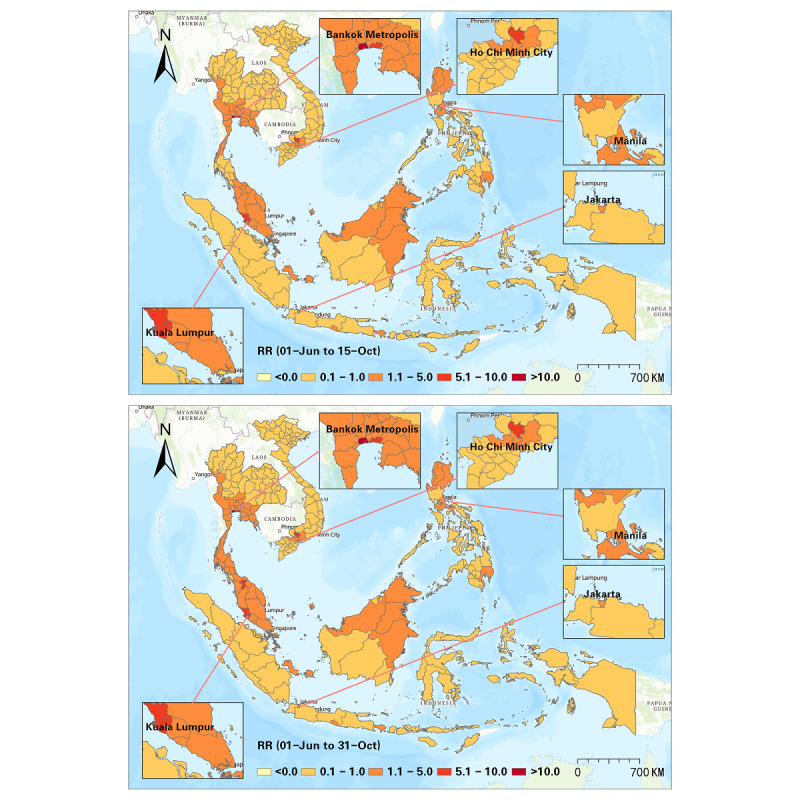
Spatial patterns of progression of the COVID-19 RR in SEA (June 1-October 15 and June 1-October 31, 2021). RR: relative risk; SEA: Southeast Asia.

Furthermore, we detected progression of the RR at a half-month interval, and a sum of 10 intervals was used to illustrate the progression of the Delta variant from June to October 2021 (Figures 5-9). In the first half of June 2021, elevated risks were identified in the middle south of Thailand, many states in Malaysia, and many districts in the Philippines, while 70% (44/63) of the districts in Vietnam manifested RR=0 during this period (Figure 5). These patterns revealed that the potential new wave of the pandemic was more likely to emerge in those high-risk districts. From then on, the Delta variant spread in SEA, and the capital areas of several countries were severely affected in SEA (eg, Bangkok Metropolis and surrounding areas, Kuala Lumpur and surrounding areas, Jakarta and surrounding areas). On the contrary, the RR in the Manila capital region in the Philippines decreased (Figure 6). By the middle of August 2021, Thailand had been influenced by the expansion of COVID-19, and most districts showed an elevated risk. Additionally, most high-risk areas in the previous months remained severe during this time, although the RR of some districts slightly declined (eg, Bangkok, Samut Sakhon, Jakarta, Riau, Sarawak). This phenomenon also reflected a high infection capability of the Delta variant (Figure 7). In the next 1.5 months (ie, August 15-September 30, 2021), the situation in Indonesia improved. North Philippines, however, showed an increased RR on September 15, 2021 (Figures 7 and 8). In the next month, the RR of Singapore increased from 0.44 to 1.53, and the RR of northern Malaysia and southern Thailand worsened as well (Figure 9). Up to October 31, 2021, the situation of Thailand, Malaysia, Singapore, Ho Chi Minh City in Vietnam, and capital areas in the Philippines was still alarming, and further studies should focus on this.

## Discussion

### Principal Findings

In this study, we utilized prospective space-time scan statistics to detect the emerging and existing space-time clusters of COVID-19 in SEA. We found that most districts in Malaysia and the Philippines, Ho Chi Minh City and Binh Duong in Vietnam, the capital and its surrounding areas in Thailand, and Indonesia exhibited a high risk of COVID-19 transmission in the early phase (June-August 2021). Space-time clusters and the RR of districts changed along with the dynamics of government interventions implemented by each country after August 2021. Indonesia mitigated the risk of pandemic transmission throughout the study period. This may be attributed to the implemented and extended the *Pemberlakuan Pembatasan Kegiatan Masyarakat* (PPKM), including policies of mandatory working from home, guidance on online teaching, and restrictions of dine-in, social gathering, and interprovince and international traveling. For example, characterized as the largest city nationwide with a high population density, Jakarta’s success in preventing exacerbation of the risk impact of COVID-19 is most likely to be related to its persistent restrictions and unchanged strategy toward COVID-19 [55].

In most of the countries where restrictions were not consistent, the RR showed an increase after they loosened restrictions. For instance, the Philippines imposed border and movement restrictions, including the strictest level of lockdown and online teaching, that managed to mitigate the pandemic RR between June and August 2021. In contrast, the Manila capital region temporally eased the movement restriction from August 21 to 31, 2021; reopened restaurants and churches; and replaced a large-scale coronavirus restriction from September 15, 2021. The results showed that the RR in the capital regions of the Philippines elevated thereafter, and the gradually intensified risks of districts in the Philippines should receive continuous attention. Particularly, although a series of regulations and restrictions, including lockdowns, curfews, and social distancing measures, had been specifically implemented in Ho Chi Minh City, the city still manifested overall worsen symptoms. This may be related to the high contagiousness of the Delta variant and overloaded health care system in Ho Chi Minh City [56]. In addition, Ho Chi Minh City has a larger population with a more economic development level than other districts, which resulted in more infections via contact with a relatively larger number of crowds. It has been suggested that population density and contact intensity are the main drivers for the propagation and amplification of this virus [57]. Moreover, the continuous high risk in Malaysia was probably because the government gradually adopted more loose measures than the other countries in SEA, with its phase development of the National Recovery Plan since the beginning of our study period. Singapore began intermittently relaxing social restrictions since early August 2021, and the RR showed an increase since September 2021.

### Comparison With Prior Work

To the best of our knowledge, this is the first attempt to use prospective space-time scan statistics to explore the space-time progression of the COVID-19 Delta variant outbreak in SEA, as well as summarize the potential linkage between the epidemic dynamics and public health interventions. Prior studies have adopted methods including the time-series forecasting model and the modified susceptible exposed infected and recovered (SEIR) model to investigate the risk of COVID-19 propagation [58,59]. Consistent with the findings of these studies, our study proved that appropriate nonpharmaceutical interventions (NPIs) are an effective way to mitigate the transmission of COVID-19, especially in Indonesia, which implemented multiple interventions (PPKM). In contrast, loosened restrictions may increase human mobility and further raise the risk of COVID-19 transmission [60,61]. Our results indicated that easing restrictions could lead to long-term existence of high-risk clusters and recurrent high risk in certain districts. Furthermore, previous studies on modeling linkages between NPIs and COVID-19 transmission were generally retrospective or predictive analyses and often focused on a single country [62-66]. The advantage of this study over prior works is that we provided a novel insight for timely and cross-nation surveillance of the dynamics and characteristics of the Delta variant at the district level in SEA. The perspective space-time scanning method used in this study can help detect spatiotemporal dynamics of COVID-19 propagation after the implementation of interventions in real time, which is beneficial for adjusting interventions and preventing COVID-19 transmission in time.

Moreover, previous studies have indicated that the propagation of COVID-19 was influenced by diverse factors. For example, population density, human movement, and environmental factors are proven to positively influence the spread of COVID-19 [67-69]. In addition, a severe COVID-19 outbreak is more likely to occur in regions with poor socioeconomic status [70-72]. Despite this, our study further emphasized the importance of public health interventions. Hence, we suggested that continuous strict restrictions are beneficial for epidemic control, especially for developing regions with weak public health systems and relatively low vaccination rates. Furthermore, to better understand the progression of COVID-19 transmission, the detection of space-time clusters could be adjusted with covariates, such as income, age, air quality, and vaccination status, which could improve evaluation of COVID-19 transmission [16,73].

### Implications and Recommendations

Public health interventions play an important role in epidemic containment, in which social restriction policies effectively mitigate the propagation of COVID-19 [37]. Restrictions of mass gathering and travel, keeping a social distance, and reducing human mobility are beneficial to control COVID-19 because these measures can reduce the probability of exposure to virus infection [74,75]. Our study discovered the potential linkage between the dynamics of COVID-19 outbreaks and interventions. This indicated that although continuously strict restrictions contribute to preventing exacerbation of the pandemic, temporary or continuous relaxation may result in acceleration of epidemic propagation. Appropriate restriction policies are key to preventing the pandemic, because high transmission of the COVID-19 variant would lead to worse situations [76]. In addition, if the number of community cases exceeds imported cases, border restrictions would be less valuable than domestic measures. In this case, authorities should emphasize more on domestic intervention in order to reduce community transmission [77]. Nevertheless, intervention measures against COVID-19 require adequate resources and good socioeconomic status. When implementing the intervention, economic and social justification is one of the priorities that governments should consider [78]. Hence, it is a challenge for all countries to weigh the balance between epidemic development and socioeconomic loss [79].

Considering domestic social and economic status, most countries in SEA gradually changed their strategies from the elimination of cases to living with COVID-19 since August 2021 [7]. A concurrent trend observed in SEA is that all countries except for Indonesia have been gradually loosening social restrictions, allowing international communications, while boosting vaccination to achieve group immunity. For instance, the Singapore Ministry of Health believes that with the assistance of a high vaccination rate, the economy and social norms could be restored without causing uncontrollable disease outbreaks or breakdown in the hospital system [80]. Vaccination is increasingly essential to protect the crowd from the exacerbating threat of morbidity and mortality of this Delta variant and future variants [81-84]. However, a previous study found that the effectiveness of available vaccines against the Delta variant (B.1.617.2) showed a reduction compared to previous virus variants [85], implying that current vaccination is likely to become ineffective against future variants [86]. Considering the fragile health systems in SEA, implementations including contact tracing, quick isolation, and strict restriction are still essential to prevent potential future outbreaks [87,88].

International coordination also plays an important role in responding to the pandemic. This includes information sharing, vaccine donation, medical support, and industry cooperation [89,90]. The Association of Southeast Asian Nations (ASEAN) countries are advised to exploit the strong socioeconomic connectivity to adopt collaborative policies in response to COVID-19. To facilitate cooperation among countries in SEA, regional surveillance is indispensable as it provides necessary information about emerging risks in the event of potential outbreaks due to new variants. This approach supports more precise prevention and mitigation of COVID-19, thus minimizing the cost of relevant resources. It was reported that Singapore and Vietnam provided medical equipment and support to neighboring countries [3], and we hope that there will be more multilateral collaborations among countries in SEA, especially considering the long-term challenges brought about by emerging new COVID-19 variants.

### Limitations

Despite the insights from our study, there are notable limitations in the COVID-19 data. To begin with, only 7 of 12 countries provided data at the primary administrative district level, so we were not able to explore the complete propagation process in SEA. In fact, many previous studies also faced a shortage or loss of available data (ie, insufficient pediatrics data) [91-93]. Additionally, although this study and many previous studies adopted COVID-19 case report data for analysis, these data may be confounded by underreporting. Due to insufficient testing data, this study did not account for different spatiotemporal screening rates [94,95]. In addition, if higher spatial resolution data were available (ie, city, county, and even block or subzone), more specific and detailed patterns could be revealed. Insufficient knowledge of the data or dynamics would lead to invalidity and unreliability of responses to COVID-19 [96]. Therefore, we strongly suggest that public health authorities in SEA should disclose more representative and reliable data [97,98]. Furthermore, although this study focused on the Delta variant of COVID-19, the data we adopted inevitably included cases from all variants. Although the Delta variant dominated the second outbreak in SEA since June 2021, this may result in uncertainty, in that proportion of cases caused by the Delta variants were not the same in different countries. Moreover, COVID-19 transmission and its impacts have shown environmental inequality in terms of household income, education level, age, gender, etc [99,100]. The potential correlation between environmental inequality and COVID-19 should be further studied using diverse data in order to obtain significant insights into resource allocation and regional prevention.

### Conclusion

The prospective space-time scan statistics revealed the potential linkages between public health interventions and the risk of the Delta variant of COVID-19 transmission. Regions that continuously adopted strict restrictions have witnessed a decreasing risk of pandemic progression, whereas some countries that implemented loosened interventions have shown a relatively higher risk. Moreover, our approach can be used to monitor the dynamics of COVID-19 with the latest data and support timely adjustments of domestic and interregional public health interventions to prevent further deterioration of the pandemic situation.

## Abbreviations

DIY: Daerah Istimewa Yogyakarta
NPI: nonpharmaceutical intervention
PPKM: Pemberlakuan Pembatasan Kegiatan Masyarakat
RR: relative risk
SEA: Southeast Asia
